# Self-Exclusion from Gambling—A Measure of COVID-19 Impact on Gambling in a Highly Online-Based Gambling Market?

**DOI:** 10.3390/ijerph18147367

**Published:** 2021-07-09

**Authors:** Anders Håkansson, Carolina Widinghoff, Jonas Berge

**Affiliations:** 1 Department of Clinical Sciences Lund, Faculty of Medicine, Lund University, 221 00 Lund, Sweden; carolina.widinghoff@med.lu.se (C.W.); jonas.berge@med.lu.se (J.B.); 2 Malmö Addiction Center, 205 02 Malmö, Sweden

**Keywords:** COVID-19, gambling disorder, problem gambling, behavioral addiction, self-exclusion

## Abstract

The COVID-19 pandemic, and related changes of the gambling market, have been suspected to affect the risk of problem gambling. Despite media attention and political concern with this risk, study findings hitherto have been mixed. Voluntary self-exclusion from gambling was introduced on a national level in Sweden as a harm reduction tool in 2019, and this self-exclusion service in Sweden is a rare example of such an official, nationwide, multi-operator system. The present study aimed to evaluate whether short-term self-exclusion patterns were affected by different phases of COVID-19-related impacts on gambling markets in 2020. During the lock-down of sports in the spring months of 2020, three-month self-exclusion was unaffected, and one-month self-exclusion appeared to increase, though not more than in a recent period prior to COVID-19. Despite large differences in sports betting practices between women and men, self-exclusion patterns during COVID-19 were not apparently gender-specific. Altogether, self-exclusion from gambling, to date, does not appear to be affected by COVID-19-related changes in society, in contrast with beliefs about such changes producing greater help-seeking behavior in gamblers. Limitations are discussed, including the fact that in a recently introduced system, seasonality aspects and the autocorrelated nature of the data made substantial statistical measures unfeasible.

## 1. Introduction

Problem gambling, including the narrower and more clearly defined diagnostic construct of gambling disorder, is known to be characterized by pronounced financial and psycho-social consequences [1]. While a unanimous definition of problem gambling is lacking, it is typically defined using screening and assessment scales with items describing gambling for more money than one can afford, the practice of coming back to gambling in order to ‘chase losses’, the health consequences of gambling, feelings of guilt and the financial consequences for one’s household [2]. Many problem gamblers do not seek formal treatment [3], and, instead, it is common for problem gamblers to turn to other online forms of harm-reducing measures, such as voluntary self-exclusion tools [4]. Voluntary self-exclusion from gambling is a prevention or harm reduction tool, where one can register with a service which prohibits oneself from entering the physical venue or the online service of a gambling operator, such that no bet can be placed. Such self-exclusion sites may be related to one specific operator, or a number of venues of online gambling services, and should, in order to be effective, involve a time period during which the voluntary ban against gambling cannot be breached. In Sweden, a rare example of a nationwide, multi-operator online self-exclusion system involving all licensed operators was introduced in 2019 [5].

The COVID-19 pandemic has been suggested to affect gambling patterns, due to changes occurring to sports events and physical gambling venues, changes in daily habits and the development of psycho-social and financial problems [6]. Recent publications have demonstrated decreasing overall gambling activity [7,8], whereas it has been suggested that some transition may occur from land-based to online gambling during physical lock-down restrictions [9]. For obvious reasons, land-based and sports-related gambling were more strongly affected by the pandemic than online chance-based games which, technically, were not impacted by stay-at-home recommendations [10,11,12]. In Sweden, problem gambling was considerably more prevalent among individuals who self-reported increased gambling during COVID-19 [10]. Altogether, while most individuals’ gambling may have decreased or been unaffected by COVID-19, potentially severe consequences in some subgroups still need to be surveyed. 

COVID-19-related changes of the gambling market affected different gambling types diversely, and to significant extent. From mid-March to mid-May 2020, major sports events and opportunities for sports betting were cancelled [13]. Gambling habits differ strongly across gender; men are considerably more likely to report sports betting and women are more likely to report betting in online casino or bingo [14,15,16,17], the latter being the gambling types less affected by COVID-19. Thus, it is possible that COVID-19-related effects on the gambling market may affect women and men differentially. In an early survey study assessing gambling during the pandemic, individuals who migrated from sports betting to other gambling types were more likely to be male [10]. Also, based on previous experience, it may be that female gambling behaviors may be more strongly associated with poor mental health [17]. 

Early studies from the present setting, carried out from April through November, 2020, indicate a possible increase in problem gambling and a possible migration of gambling towards riskier gambling types [10,11,12,13]. In addition, early media reports indicated a fear of increased treatment needs for problem gambling during the COVID-19 pandemic, with reports about a perceived interest in seeking help from peer support organizations [18]. Thus, the rationale behind the present study is that help-seeking behavior was hypothesized to have increased, and therefore, that this may also involve an increase in voluntary self-exclusion, in line with early fears expressed by stakeholders in the area. Very little is known about how self-exclusion has changed as a response to COVID-19’s changes to the gambling market. The present study aims to analyze the course of self-exclusion in Sweden’s nationwide, multi-operator self-exclusion system, across different acute phases of the COVID-19 pandemic and assessing, separately, possible trends of self-exclusion for women and men. The hypothesis was that trends in self-exclusions would be affected by the most pronounced events occurring in the world of sports during the first months of the pandemic and that these changes would be different in men and women. 

## 2. Materials and Methods

The present study is an analysis of the number of individuals who self-excluded before and during the COVID-19 pandemic in Sweden. In January 2019 Swedish legislation introduced a nationwide self-exclusion system, where any individual in Sweden can voluntarily self-exclude from all licensed gambling operators (sports and horse race betting, online casinos, state-owned land-based casinos and other chance-based games and lotteries online). Exceptions from the multi-operator nationwide self-exclusion service are the minor, limited-deposit ‘restaurant casinos’ and physical lottery tickets. The self-exclusion service (spelpaus.se), referred to in Swedish as Spelpaus (‘gambling pause’) and described in previous research [5,19], is managed by a governmental authority, independent of gambling operators. Self-exclusion is carried out online and clients choose to self-exclude either for one, three, six or twelve months. 

In the present study, data were acquired from the Swedish Gambling Authority, and describe the number of people, for each gender, self-excluded on every Monday. This weekly number was used to reflect the number of people who were enrolled as new self-excluders up to that date, including the past week and weekend. As this self-exclusion system was introduced on 1 January 2019, a large number of people registered in the very first weeks of 2019. In addition, for many of the clients self-excluding for an entire year or for half a year, these periods of time may have ended by COVID-19’s beginning, and therefore may have been extended during the first weeks of 2020. Thus, short-term changes in the gambling market are unlikely to be easily detected in the number of individuals self-excluding on a given date for the longer time intervals. Thus, in the present analyses, self-exclusion durations for one and three months were assessed, whereas data on self-exclusion for six or twelve months were not considered. 

The time frame of the study was adapted to relevant changes to the gambling market in 2020 (see Table 1). Two time points were considered to have had a COVID-19-related effect on gambling; the sports lockdown (16 March) and relief from this absence after the return of one major European soccer league (18 May). These two time points have been used descriptively in the present paper and data were collected through 16 June 2020.

The present study was reviewed by the Swedish Ethics Review Authority (file number 2020-03847), which stated that the present research, which does not involve individuals who can be identified, does not formally require ethical permission. It also expressed that it did not have any ethical concerns regarding the study. 

### Statistical Methods

Data were handled using the statistical software package R3.5.3 (R Core Team, 2009, R Foundation for Statistical Computing, Vienna, Austria). Data were presented descriptively, including numbers of female, male and total self-excluded individuals, from the start of the *Spelpaus* system (1 January 2019) until the end of the study period. Data included changes around the key dates of 16 March and 18 May 2020 (and in comparison with the first measure of the year, 6 January 2020). Statistical analyses of potential changes in self-exclusion were considered, but because of two issues with the data we decided that a formal statistical analysis of potential changes was not feasible. The first issue was the possibility of seasonality in the data, i.e., that there is a seasonal variation in gambling behaviors and thus, hypothetically, also in motivations to self-exclude from gambling services. In order to handle the effect of seasonality with time series data, there is a need for data that span several cycles (ie., whether the seasonality reflects a weekly, monthly or yearly variation). As we only have data from one full calendar year prior to the period of interest (the first 24 weeks of 2020), and based on the fact that the self-exclusion system was introduced in the beginning of 2019, the data cannot be assumed to be representative of a stabilized system. Therefore, we were unable to control for any seasonality effects. The other issue was having only group-level data for the number of individuals currently in self-exclusion at any given time, from which we could not determine the number of new individuals that chose to self-exclude for each point of observation. This introduced an extreme amount of autocorrelation, i.e., that the value of a given point of observation was highly correlated with the values of nearby points of observation (Supplementary Figure S1). We therefore constructed a new variable, weekly change, which reflected not the number of new individuals that self-excluded each week, but the net change in the number of individuals self-excluding. This reduced the problem with autocorrelation substantially, though not completely (supplementary Figure S2). In summary, we concluded that no statistically meaningful inferences from the data could be made, so we opted for a descriptive visual analysis of the data as presented below. Changes in the number of self-excluded for one- and three-month periods are reported as percentages for women and men, respectively. These changes refer to the periods of 2020 between the time points describing the start of the year (first full week, starting 6 January until the onset of COVID-19-related changes), the onset of the sports lock-down (16 March), the normalization of the sports betting market (18 May) and the end of the study (16 June). 

## 3. Results

### 3.1. One-Month Self-Exclusion 

Overall, one-month Spelpaus self-exclusion increased considerably during 2020, both before and during the sports lock-down of the pandemic. The increase in the number of self-excluded men during COVID-19 appeared to be larger than for women, but increases had been seen in both men and women prior to the pandemic. 

**Figure 1:**
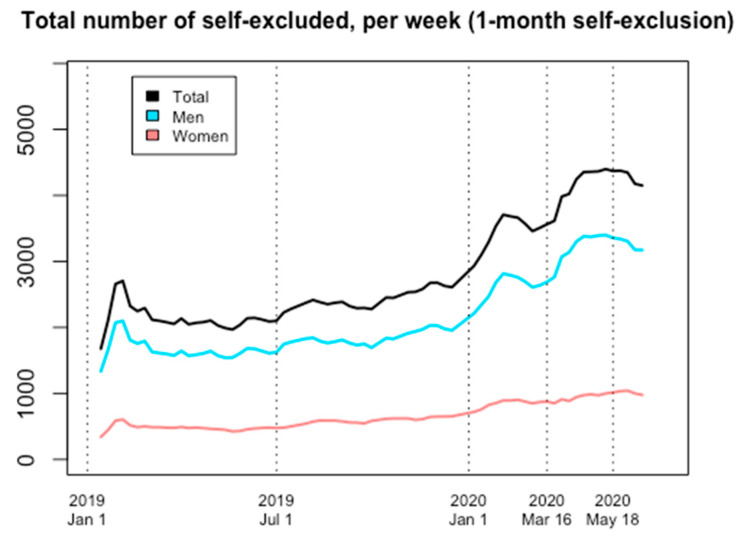
Number of individuals self-excluded for a one-month interval on every Monday, 1 January 2019 through 16 June 2020.

**Figure 2:**
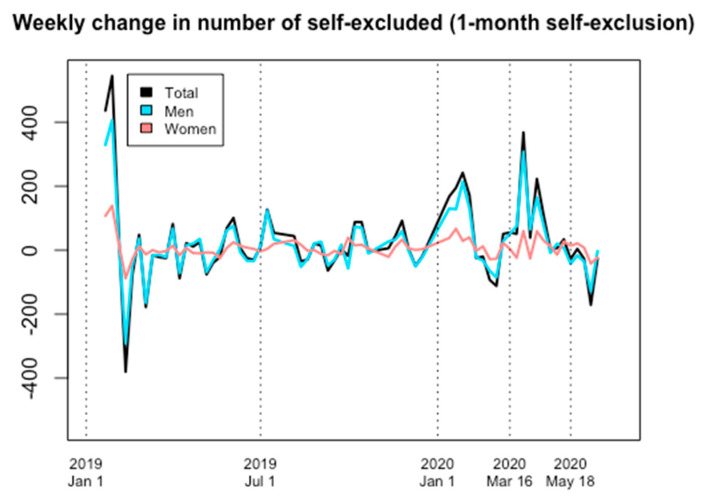
Weekly change in number of individuals self-excluded for a one-month interval, 1 January 2019 through 16 June 2020.

In more detail, the numbers of self-excluded individuals at the start of the pandemic (16 March) already represented a marked increase in comparison to the start of 2020 (6 January); 22 percent more women than on 6 January, and 22 percent more men (+22 percent for the entire group, compared to 6 January). On 16 March 2020, a total of 875 women and 2689 men had self-excluded for a one-month period (a total of 3564 individuals). At sports reopening, on 18 May 2020, these values had changed to 1013 women (+16 percent compared to 16 March) and 3357 men (+25 percent), to total 4370 individuals (+23 percent). By the end of the study, on 15 June 2020, these values had stabilized at 977 for women (+12 percent compared to 16 March) and 3173 for men (+18 percent), to total 4150 individuals (+16 percent compared to 16 March) (Figure 1 and Figure 2). 

### 3.2. Three-Month Self-Exclusion

Overall, three-month Spelpaus self-exclusion also increased markedly during 2020, with such an increase occurring prior to the onset of the pandemic. No obvious increase in the number of male self-excluders occurred during COVID-19, and although a modest increase was seen in women, these changes were more pronounced prior to the pandemic (6 January to 16 March). 

**Figure 3:**
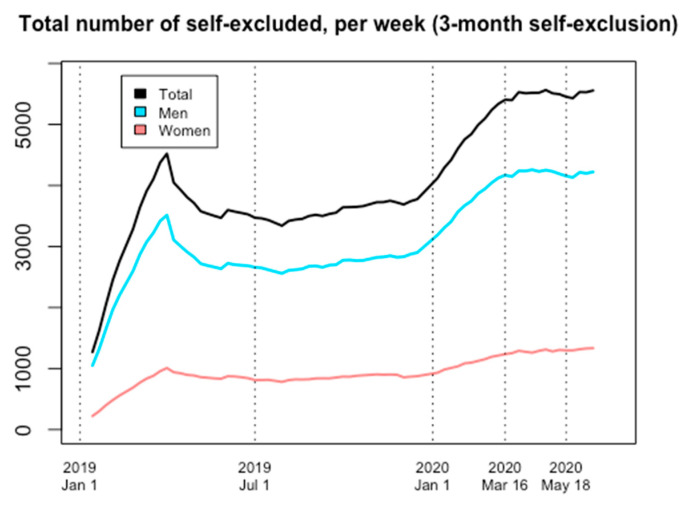
Number of self-excluded on every Monday, 1 January 2019 through 16 June 2020. Self-exclusions with a three-month duration.

**Figure 4:**
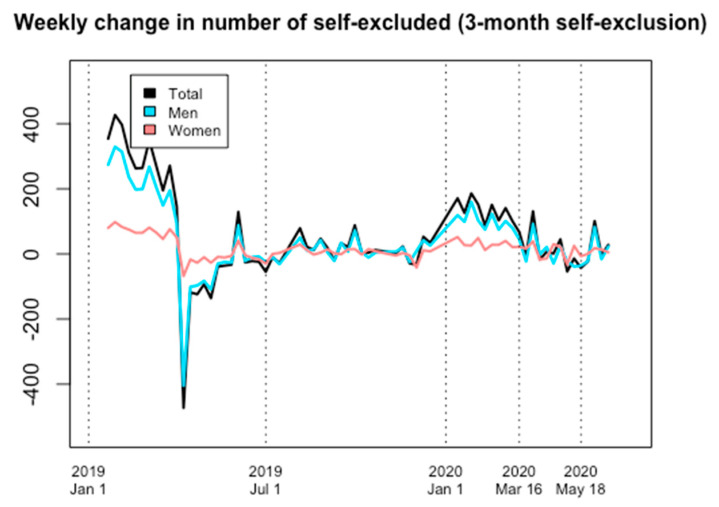
Weekly change in number of self-excluded, 1 January 2019 through 16 June 2020. Self-exclusions with a three-month duration.

The number of self-excluders on 16 March represented a marked increase in comparison to the start of 2020 (6 January); 33 percent more women were in self-exclusion than had been on 6 January, as were 31 percent more men (+31 percent in total). With respect to the three-month self-exclusion service, the total number of self-excluded women and men on 16 March 2020 were 1236 and 4169, respectively (total 5405). At the reopening of sports, on 18 May 2020, the corresponding numbers were 1298 women (+5 percent compared to 16 March) and 4155 men (+/−0 percent), in total 5453 (+1 percent); at the end of the study, on 16 June, 1334 women (+8 percent compared to 16 March) and 4222 men (+1 percent) were self-excluded, a total of 5556 (+3 percent compared to 16 March) (Figure 3 and Figure 4). 

## 4. Discussion

The present study is the first to analyze self-exclusion rates as a description of gambling-related behavior during COVID-19. Theoretically, these could differ between genders, given the uneven impact of COVID-19 on different gambling types which are often preferred differentially by women and men. The present study did not clearly demonstrate such effects. For both genders, and for both self-exclusion intervals studied, a pronounced increase was already evident during the months immediately prior to the onset of COVID-19, whereas the continued increase during the changes occurring in the gambling market were less pronounced. Also, during COVID-19 no increase was seen in men for the longer self-exclusion period, whereas a modest increase was seen in women, and for the shorter self-exclusion period, modest increases were seen in both genders. However, in both cases, increases had already been pronounced for both self-exclusion periods during the last months prior to COVID-19. Thus, three-month self-exclusion did not demonstrate any obvious COVID-19-related effect, and for one-month self-exclusion, an increase could be displayed graphically during the sports lock-down period, although with no convincing gender differences, and these changes were not clearly more pronounced than the increase occurring prior to the pandemic. Thus, although reliable statistical analyses were unfeasible, despite attention to gambling during COVID-19 in political discussions and in the media, and although COVID-19-related changes in gambling in the general public cannot be excluded, such changes did not clearly translate into self-exclusion enrollment during the first few months of the pandemic. 

If rates of self-exclusion were assumed to change in response to the perturbations of the gambling market during the pandemic, such a development was assumed to be different in women and men. Among treatment-seeking patients of a treatment facility in the present setting, in a previous study around half of men, but virtually no women, reported problematic sports betting [16]. In survey data from online moderate-risk gamblers, men were significantly more likely to report sports betting, whereas women were significantly more likely to report online casino gambling [17]. Thus, effects on self-exclusion from the nearly total lock-down of sports events would likely affect male self-exclusion behavior more than in women; however, this was not apparent here. 

Despite the limitations of statistically assessing the data (which were mostly due to the limited comparison time prior to the pandemic) the lack of a clear association with COVID-19 stands in contrast to early media reporting of impressions that peer support gamblers’ groups were seeing increased help-seeking behavior during COVID-19. However, such impressions have not been quantified systematically. In addition, recent data from the present setting demonstrated that not all self-excluders are problem gamblers, and vice versa—many problem gamblers are not self-excluded [5,19]. Also, it should be borne in mind that individuals with a gambling disorder may also have perceived the early parts of the pandemic as relieving, if their gambling problem was primarily related to gambling types which were unavailable, therefore possibly facilitating abstinence in these individuals [20]. Likewise, in previous survey studies in the present setting, the subgroup of gamblers who reported a decrease in gambling during COVID-19 was, in size, comparable to the proportion reporting an increase. Thus, the absence of dramatic changes in treatment seeking [21] or self-exclusion may be a result of counter-acting changes in different subgroups of gamblers, reflecting the mixed picture of COVID-19’s impact on gambling as either concerning or, paradoxically, relieving [20]. 

Also, this is, to the best of our knowledge, the first scientific paper to describe short-term enrolment in the nationwide, multi-operator self-exclusion system for gambling in Sweden. From the present graphical descriptions, it appears that gambling self-exclusion may have received gradually increasing attention since this service was introduced in early 2019. This merits further study assessing how gambling problems may have been affected in Sweden by the introduction of this nationwide, multi-operator and thereby rare, self-exclusion service, both throughout the pandemic and into post-COVID phases where the financial and psychological consequences of the pandemic may persist. It should be borne in mind, however, that self-exclusion is likely to reflect the population of problem gamblers only partly; point prevalence estimates indicate that around 30,000–40,000 individuals are likely to meet the criteria for a gambling disorder in Sweden, and more than 100,000 may be problem gamblers [2]. In total, including also the six- and twelve-month exclusion periods, more than 50,000 Swedes are reported to have self-excluded via the Spelpaus system since it began [19], but recent data have demonstrated that a substantial number of self-excluders may not be problem gamblers, and likewise, many problem gamblers are likely found among non-excluded gamblers [5]. Thus, numbers of self-excluders may reflect problems or concerns about gambling in a broader sense than a separate individual’s gambling problems, and may occur due to an aversion to gambling advertising or because of an individual’s historical gambling problems [5].

The present study has limitations; although it assesses a nationwide, multi-operator self-exclusion service, it relates to only one specific geographical setting. In addition, Sweden is regarded to have applied strategies for COVID-19 prevention different from those in many other countries, for example having forgone formal lockdown procedures for general society [22]. However, this limitation can itself be considered limited, due to the fact that a large part of the impact on gambling markets in the spring months of 2020 was global; sports events available for gambling were cancelled globally (and in Sweden) and thus our analysis does not only refer to sports cancellation within-setting. Likewise and also in Sweden, other land-based gambling types were reduced or frequented less during the first months of the pandemic, as shown in early survey data [10] and in revenues of gambling operators [13]. Another limitation is the time frame of study, due to effects likely related to the relative novelty of the Spelpaus system, clear periods of comparison during a non-affected year were not available for statistical comparison. These seasonal patterns were far too likely to have an impact on our findings, given that an overall increase in self-exclusion in this relatively novel system had happened before, particularly during the months immediately before Sweden’s COVID-19 outbreak. For example, it cannot be excluded that individuals who self-excluded early in the system’s introduction in January 2019 may have extended their shorter self-exclusion periods repeatedly, producing an incidental increase occurred specifically at the start of 2020. As described above, even for the shorter self-exclusion periods, patterns of possible seasonality and the recency of the initiation of the system (around 14 months prior to the pandemic) limited the possibility of conducting formal statistical analyses. Moreover, it is also insufficiently understood from previous Spelpaus data whether the choice of a longer self-exclusion period is associated with a different clinical picture of problem gambling severity, a topic which merits further research [19]. Overall, the facts that data were aggregated and that identified individuals’ age, gambling patterns and longitudinal course of self-exclusions could not be followed also represented a limitation. 

In contrast to the limitations addressed above, the present paper, on the other hand, has the merit of being the first scientific description of self-exclusionary gambling patterns during the COVID-19 crisis, and given the nationwide, multi-operator nature of Sweden’s self-exclusion system, it can uniquely demonstrate self-exclusion behaviors in the population of an affecting system which is unrelated to specific gambling operators or venues, and potentially more likely to represent a help-seeking behavioral drive in a broader sense. Also, based on the nature of the Spelpaus system, the sample size has the strength of being larger than could be expected from a more local or operator-specific system. 

## 5. Conclusions

In conclusion, short-term national multi-operator self-exclusion from gambling did not clearly appear to be affected by the dramatic societal changes in the early phases of the COVID-19 pandemic; despite the clear gendered differences between the gambling types affected, gendered differences in self-exclusion patterns were not clearly demonstrated. Increases in the number of new self-excluders during sports lock-down were either absent or modest and lower than in the months preceding the pandemic. Given the recency of such self-exclusion systems, longer time frames will be needed in order to outline the full impact of the pandemic on gambling and self-excluding behavior. In addition, whether or not a person self-excludes from gambling may not be a direct effect of changes in the gambling market, but, possibly and moreover, due to long-term negative trends in their household economy and mental health. Further research in this area needs to assess the reasons for self-excluding from gambling during the pandemic. Gambling as a public health concern warrants further study in relation to COVID-19’s impact on mental health, and, there, more long-term studies of self-exclusion patterns may serve as a viable data source. 

## Figures and Tables

**Table 1:** Major events representing the impact of COVID-19 on sports betting and land-based casino gambling in Sweden during early phases of the pandemic in 2020.

Date	Event with Impact on Sports Betting and Land-Based Gambling Market
9 March	Announcement of the interruption of the Italian soccer league.
15 March	Swedish ice hockey league postponed.
19 March	Higher Swedish soccer leagues postponed.
1 April	Closing of the four state-owned major casinos in Sweden.
8 April	Swedish government presents suggested regulations to the gambling market in order to decrease consequences of the pandemic (and thereby highlight possible risks of gambling during COVID-19).
9 May	Opening of the Korean soccer league.
16 May	Opening of the German soccer league.
29 May	Announcement that the Swedish soccer league will restart on 14 June.
14 June	Opening of the Swedish soccer league.
17 June	Opening of the English soccer league.

## Data Availability

Data can be obtained freely upon request to the first author.

## Supplementary Materials

The following are available online at https://www.mdpi.com/article/10.3390/ijerph18147367/s1, Figure S1: Autocorrelations for the total numbers of self-excluded, per week, for the 1-month and 3-month self-exclusion periods, respectively. Figure S2: Autocorrelations for the weekly changes in numbers of self-excluded, per week, for the 1-month and 3-month self-exclusion periods, respectively.
