# Supplementary material for: Self-Exclusion from Gambling—A Measure of COVID-19 Impact on Gambling in a Highly Online-Based Gambling Market?
Source: Int J Environ Res Public Health. 2021 Jul 9;18(14):7367. doi: 10.3390/ijerph18147367 (PMC8305751; doi:10.3390/ijerph18147367)

Supplementary Figure S1. Autocorrelations for the total numbers of self-excluded, per week, for the 1-month and 3-month self-exclusion periods, respectively.

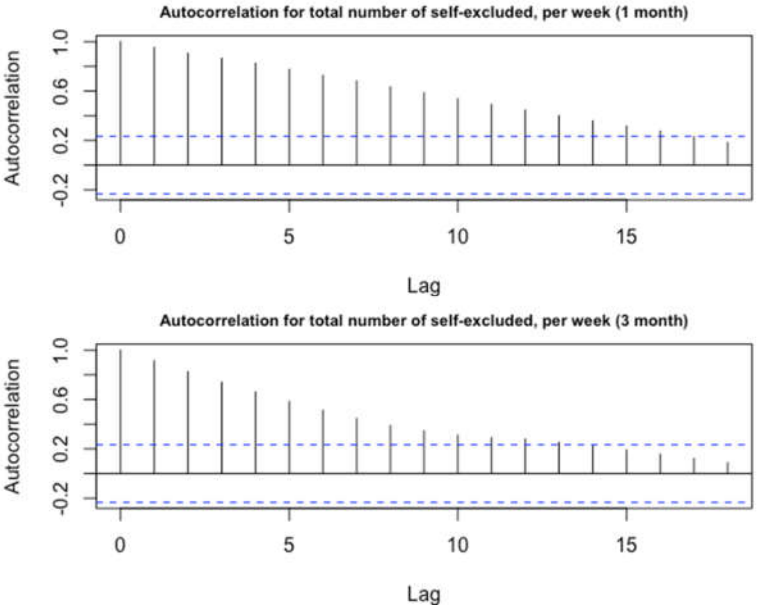

Supplementary Figure S2. Autocorrelations for the weekly changes in numbers of self-excluded, per week, for the 1-month and 3-month self-exclusion periods, respectively.

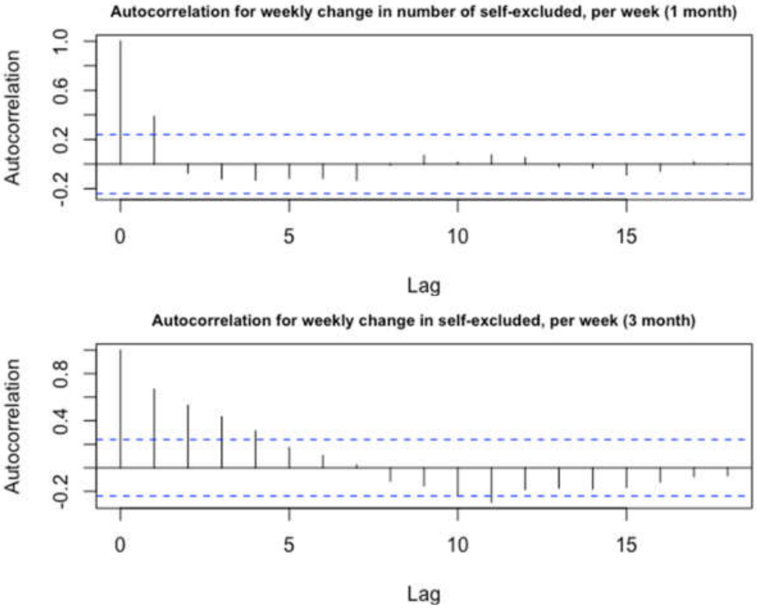

Supplement: Supplementary file 1 [file ijerph-18-07367-s001.zip › ijerph-1279234-supplementary.pdf]
